# Enhanced Peroxidase-Like and Antibacterial Activity of Ir-CoatedPd-Pt Nanodendrites as Nanozyme

**DOI:** 10.1155/2023/1689455

**Published:** 2023-02-15

**Authors:** Jingfang Song, Jian He, Lin Yang, Weiguo Wang, Qinqin Bai, Wei Feng, Ranhui Li

**Affiliations:** ^1^The Second Affiliated Hospital, Department of Pediatrics, Institute of Pathogenic Biology, Hengyang Medical School, University of South China and Hunan Provincial Key Laboratory for Special Pathogens Prevention and Control, Hengyang, Hunan 421001, China; ^2^Institute of Pharmacy and Pharmacology, Hengyang Medical School, Hunan Province Cooperative Innovation Center for Molecular Target New Drug Study, University of South China, Hengyang, Hunan 421001, China; ^3^Department of Public Health Laboratory Science, School of Public Health, Hengyang Medical School, University of South China, Hengyang, Hunan 421001, China; ^4^Institute of Pathogenic Biology, Hengyang Medical School, University of South China and Hunan Provincial Key Laboratory for Special Pathogens Prevention and Control, Hengyang, Hunan 421001, China; ^5^College of Basic Medical Sciences, Youjiang Medical University for Nationalities, Baise Guangxi 533000, China

## Abstract

To inhibit the growth of bacteria, the DA-PPI nanozyme with enhanced peroxidase-like activity was synthesized. The DA-PPI nanozyme was obtained by depositing high-affinity element iridium (Ir) on the surface of Pd-Pt dendritic structures. The morphology and composition of DA-PPI nanozyme were characterized using SEM, TEM, and XPS. The kinetic results showed that the DA-PPI nanozyme possessed a higher peroxidase-like activity than that of Pd-Pt dendritic structures. The PL, ESR, and DFT were employed to explain the high peroxidase activity. As a proof of concept, the DA-PPI nanozyme could effectively inhibit *E. coli* (G^−^) and *S. aureus* (G^+^) due to its high peroxidase-like activity. The study provides a new idea for the design of high active nanozymes and their application in the field of antibacterial.

## 1. Introduction

Over the last two decades, the increasing spread of bacterial infections in medical community led to high mortality and morbidity and became a serious problem worldwide [1]. Antibiotics were the most widely used drugs to treat the bacterial infections and saved countless human lives. However, the overuse of antibiotics leads the bacteria such as *Staphylococcus aureus* (G^−^) and *Escherichia coli* (G^+^) to be multidrug-resistant, which turned out to be a major threat to hospitalised or immunocompromised patients [2]. Therefore, the new effective and safe drugs to eradicate bacterial infections need to be developed.

Enzyme is an indispensable core substance of life. Many key technologies and product manufacturing, such as in bionics [3], environmental treatment [4], antibacterial [5], and biomedical applications [6], are inseparable from enzymes. However, the applications in these areas of enzymes are limited by the low stability, time-consuming preparation, and purification. Nanozymes are inorganic nanomaterials that possess the enzyme-like activity (such as peroxidase-like activity). Compared to the nature enzyme, nanozymes possess the advantage of easy preparation, adjustable activity, and high stability. Thus, nanozymes have the potential to replace biological enzymes. The peroxidase-like activity is widely used in fields of biomarkers detection [7], antitumor [8], antibacterial, and antiviral [9]. However, the low activity has become a bottleneck limiting the wide application of nanozymes.

After decades of development, significant progress has been made in the activity optimization of nanozymes. Jiang et al. [10] found that the smaller the particle size, the higher activity of the Fe_3_O_4_. However, the stability of the nanozymes will decrease while the particle size is reduced to a certain size (<2 nm). Nowadays, the structures with high specific surface area (porous and dendrites) have been applied to enhance the activity of nanozymes. For example, Chen et al. [11] found that porous materials can enhance the peroxidase-like activity of nanozymes. The Pt-Pd porous nanorods showed a higher peroxidase-like activity than that of Pd-Pt nanorods and Pd-Pt nanoparticles [12]. In contrast, dendritic structures have attracted extensive attention due to the large specific surface area, high mass transfer efficiency, and a large number of low-coordination atoms. Ge [13] found that branched structures with high density of active site can enhance the activity of Pt hollow nanodendrites.

In the previous reports, the branch ends of the dendritic structure are mostly monometallic. The peroxidase-like activity of monometallic nanozyme is always weaker than that of multimetallic nanozyme [14, 15]. The Sabatier principle states that the catalyst-intermediate interaction should be moderate [16, 17]. The alloying is an appropriate way to adjust the interaction between the surface of catalyst and intermediate. For example, the Au doped Pd two-dimensional nanosheet showed a higher peroxidase-like activity compared to Pd nanosheet [18]. Among the transition metal, thanks to the high affinity to H_2_O_2_, iridium (Ir) is always used to form alloy to enhance the peroxidase-like activity of nanozymes [19]. However, the iridium is rarely applied to form alloy on nanodendrites nanozymes.

Here, the Ir was used to coat on the branch ends of Pd-Pt nanodendrites (named D-PP nanozyme) to form alloyed Pd-Pt-Ir nanozyme (named DA-PPI nanozyme). The DA-PPI nanozyme shows a higher peroxidase-like activity than that of D-PP nanozyme. The DA-PPI nanozyme was finally applied in the inhibition of *E. coli* (G^−^) and *S. aureus* (G^+^).

## 2. Materials and Methods

### 2.1. Chemicals

Potassium chloroplatinate (K_2_PtCl_6_, 98%), potassium tetrabromopalladate (K_2_PdBr_4_, 98%), sodium hexachloroiridate hydrate (Na_3_IrCl_6_·xH_2_O, 99%), Pluronic F127, ascorbic acid (AA, 99%), potassium bromide (KBr, 99%), terephthalic acid (TA), poly (vinyl pyrrolidone) (PVP, MW ≈ 55,000), ethylene glycol (EG, 99%), and 3,3′,5,5′-tetramethylbenzidine (TMB) were all ordered from Aladdin.

### 2.2. The Preparation of D-PP Nanozyme

The solution (5 mL) containing different amount of K_2_PdBr_4_ and K_2_PtCl_6_ was firstly prepared. Pluronic F127 (0.05 g) was then dissolved into the above solution. The reducing agent (ascorbic acid, 0.1 M, 5 mL) was injected into the above mixture. The mixture was incubated at 30°C for 12 h. During the synthesis process of Pd-Pt nanodendrites (D-PP nanozyme), the mixture turned from brown to black. Finally, the D-PP nanozyme was obtained through centrifugal washing and stored in 10 mL ethylene glycol.

### 2.3. The Preparation of DA-PPI Nanozyme

First, about 1 mL D-PP nanozyme solution was injected into a flask with 9 mL EG. Then, the ascorbic acid (100 mg) and PVP (60 mg) were added into the mixture. The mixture was heated up to 110°C and incubated for 30 min. After the incubation, the mixture was heated up to 180°C. The Na_3_IrCl_6_ EG solution (1.5 mL, 0.15 mg ml^−1^) was slowly injected into the mixture. After the injection, the mixture was cooled down to the room temperature. The DA-PPI nanozyme was obtained through centrifugal washing with acetone once and deionized water three times. The obtained DA-PPI nanozyme was stored in 1 mL deionized water for next use. The concentration of DA-PPI nanozyme was detected to be 18.6 mgL^−1^ (detected as Pd) using ICP-MS. The preparation of DA-PPI can be summarized as the following chemical equation:

(1) $$\begin{array}{c} K_{2}PdBr_{4}\left(c_{1}\right)+K_{2}PtCl_{6}⟶PP\left(c_{1}\right)\text{,}\\ PP\left(c_{1}\right)+Na_{3}IrCl_{6}⟶PPI\left(c_{2}\right)\text{.} \end{array}$$

The yield (*y*) was calculated as the following equation:

(2) $$\begin{array}{c} y=\frac{c_{1}}{c_{0}}\times \frac{c_{2}}{c_{1}}\text{,} \end{array}$$

(3) $$\begin{array}{c} c_{1}=\frac{0.05mmol}{10mL} \end{array}$$

(4) $$\begin{array}{c} c_{2}=\frac{18.6mg/L}{106}\\ =0.175mM\text{.} \end{array}$$

Thus, the *y* is calculated to be 3.5% according to the equations (1)–(3).

### 2.4. Peroxidase-Like Activity and Kinetic Assay of DA-PPI Nanozyme

The peroxidase substrate (TMB) can be oxidised by H_2_O_2_ with the catalyst of HRP. The nanozymes possess the similar activity to HRP (peroxidase-like activity). Thus, the peroxidase-like activity of DA-PPI nanozyme is assessed by the catalytic oxidation of the TMB in the presence of H_2_O_2_. The oxidation product of TMB has an obvious UV absorption peak at 652 nm. The molar extinction coefficient (*ε*) is 3.9 × 10^4^ M^−1^ cm^−1^. The buffer is acetate buffer with pH value of 4.0. The amount of DA-PPI nanozyme used in this part is 10 *μ*L (18.6 mg/L). During the measurement of enzyme kinetic parameters of DA-PPI nanozyme towards H_2_O_2_, the concentration of TMB is fixed at 20.80 mM. The concentration of H_2_O_2_ ranged from 1.04 to 33.33 mM. The absorbance at 652 nm of different groups was recorded in 3 minutes. Similarly, when measuring the enzyme kinetic parameters of DA-PPI nanozyme towards TMB, the concentration of H_2_O_2_ was fixed at 62.5 mM. The concentration of TMB ranged from 0.10 to 6.67 mM. The parameters such as *K*_*m*_ and *V*_max_ are calculated by the following equations:

(5) $$\begin{array}{c} v=\frac{∆A}{∆t}\times \varepsilon l\text{,}\\ v=\frac{v_{\max}\times \left[s\right]}{k_{m}}+\left(k_{m}+\left[s\right]\right)\text{,} \end{array}$$

where [*s*] is the substrate concentration, *v* is the initial reaction rate, Δ*A* is the change of absorbance, Δ*t* is the change of time, *ε* is the absorbance coefficient of the substrate oxidized TMB, which is usually 39000 M^−1^ cm^−1^ at 652 nm, and *l* is the path length of light through the colorimetric dish (cm).

### 2.5. The Cytotoxicity Test of DA-PPI Nanozyme

The cytotoxicity test was performed on HeLa cells in this part. The HeLa cells were seeded in 96-well plates at 5000 cells/well and incubated 24 h. The cells were treated with final concentrations of DA-PPI nanozyme (60 *μ*g L^−1^) or DA-PPI nanozyme (60 *μ*g L^−1^) with 0.5 mMH_2_O_2_ for 12 h. The cell culture supernatants were collected and the LDH releases were detected by LDH assay kit according to the instructions of manufacturer. The experiments were repeated three times.

### 2.6. The Bacteriostasis Experiment of DA-PPI Nanozyme

The *E. coli* (G^−^) and *S. aureus* (G^+^) were selected to test the antibacterial activity of DA-PPI nanozyme using the survival ratio method. The bacteria were cultivated in LB medium at 37°C and 200 rpm for 12 h. Then, the bacterial solution was diluted to 10^6^ CFU/mL with PBS for subsequent experiments. The antibacterial experiments were divided into four groups: PBS, H_2_O_2_, DA-PPI nanozyme, and DA-PPI nanozyme + H_2_O_2_. The final concentrations of DA-PPI nanozyme and H_2_O_2_were 60 *μ*g L^−1^and 0.5 mM. The total volume of sample solution in each treatment group was 200 *μ*L. After 3 h of incubation, the solutions were gradient dilution and 20 *μ*L of the solution was placed on a solid medium and cultured for 12 h before counting the number of the bacterial colonies. And the survival ratio was calculated according to the following formula:

(6) $$\begin{array}{c} Survival\;rational=\frac{{CFU}_{experimnt}}{{CFU}_{PBS}}\times \%\text{.} \end{array}$$

## 3. Result and Discussion

### 3.1. The Characterization of DA-PPI Nanozyme

**Figure 1:**
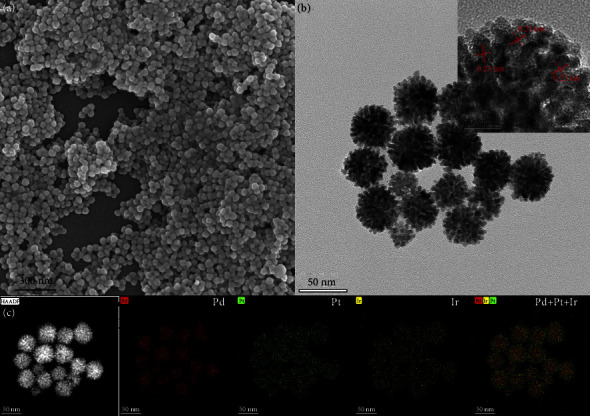
The SEM pattern of DA-PPI nanozyme (a). The TEM and HRTEM (inset pattern) pattern of DA-PPI nanozyme (b). The element distribution of DA-PPI nanozyme (c).

In this part, the morphology of DA-PPI nanozyme was characterized by a scanning electron microscope (SEM). In Figure 1(a), the DA-PPI nanozyme is nearly spherical with a uniform morphology. Subsequently, the morphology of the DA-PPI nanozyme was characterized by transmission electron microscopy (TEM). The DA-PPI nanozyme had an urchin-like structure with a large number of gaps at the branch ends providing a guarantee for the high catalytic activity (Figure 1(b)). In order to confirm the composition of DA-PPI nanozyme, the element distribution was characterized by X-ray spectroscopy (EDX). The results present that the Pd and Pt are distributed in the core and shell, respectively. The Ir is distributed on the surface of DA-PPI nanozyme. The results indicate that the elements in DA-PPI nanozyme are distributed similar to a sandwich. An alloy composed by Ir and Pt may form on the surface of DA-PPI nanozyme.

**Figure 2:**
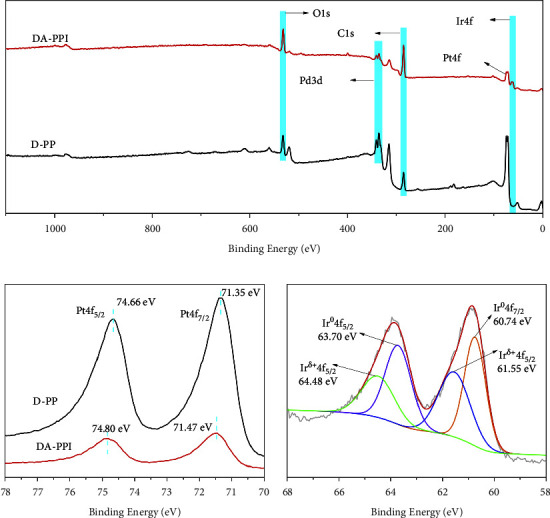
The XPS broad spectrum of D-PP nanozyme and DA-PPI nanozyme (a). The XPS narrow spectrum of Pt in D-PP nanozyme and DA-PPI nanozyme (b). The XPS narrow spectrum of Ir in DA-PPI nanozyme (c).

According to the EDX result, the DA-PPI nanozyme is composed by Pd, Pt, and Ir. However, the combination style of these three elements is not clear. Thus, the X-ray photoelectron spectroscopy (XPS) was employed to characterize the valence state changes of each element in D-PP nanozyme and DA-PPI nanozyme. The corresponding results are shown in the figure (Figure 2). In Figure 2(a), it can be seen that D-PP nanozyme contains not only Pd and Pt but also exogenous oxygen and carbon elements. Similar to D-PP nanozyme, DA-PPI nanozyme contains several elements of Pd, Pt, O, and C. What is more, the Ir can be observed in DA-PPI nanozyme according to the XPS broad spectrum. In the narrow spectrum of Pt-Pt, the Pt is split to Pt4f_5/2_ and Pt4f_7/2_. The corresponding peaks are located at 74.66 eV and 71.35 eV, respectively. In contrast, the Pt4f_5/2_ and Pt4f_7/2_ in DA-PPI nanozyme are located at 74.80 eV and 71.47 eV, respectively. After the deposition of Ir on the surface of D-PP nanozyme, the binding energy of Pt shifts to high fields. The results indicate that the chemical bond between platinum and iridium was formed during the deposition. The result is consistent with the previous report [20]. Furthermore, the narrow spectrum of Ir in DA-PPI nanozyme was analyzed. The results present that the Ir element contains Ir^0^ and Ir^*δ*+^(Figure 2(c)). The Ir^0^is split to Ir^0^4f_5/2_ and Ir^0^4f_7/2_. The corresponding peaks are located at 63.70 eV and 60.74 eV, respectively. The splitting peaks of Ir^*δ*+^(namely, Ir^*δ*+^4f_5/2_ and Ir^*δ*+^4f_7/2_) are located at 64.48 eV and 61.55 eV, respectively. This result is consistent with the previous report about the Ir characteristic peak [21].

### 3.2. The Peroxidase-Like Activity Assessment of DA-PPI Nanozyme

**Figure 3:**
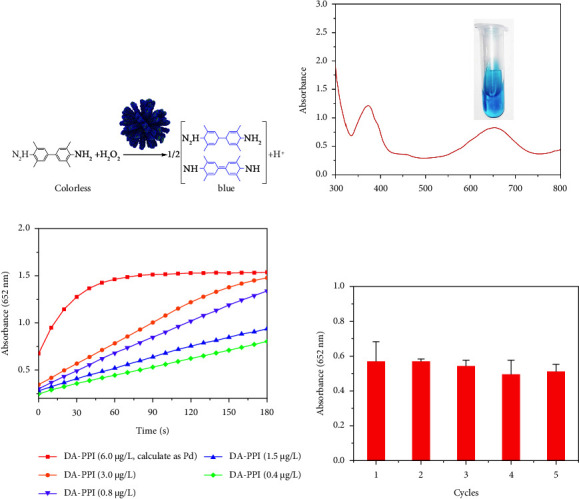
The peroxidase-like activity of DA-PPI nanozyme (a). The UV-vis spectrum of the TMB oxidative product (b). The intensity (652 nm) ranged with the concentration variation of DA-PPI nanozyme (c). The stability test for DA-PPI nanozyme in five cycles (d).

DA-PPI nanozyme can catalyze the oxidation of TMB with the participation of hydrogen peroxide (Figure 3(a)). The oxidation product is blue with obvious UV absorption at 652 nm (Figure 3(b)). The intensity reflects the peroxidase-like activity of the nanozyme. During the catalytic process, the absorbance at 652 nm increased with the increase of time. By adjusting the concentration of DA-PPI nanozyme (0.4–6.0 *μ*g/L), the absorbance at 652 nm increased with the increase of concentration in 3 min (Figure 3(c)). To evaluate its stability, the DA-PPI nanozyme was applied for catalyzing the oxidation of TMB for five cycles. The result shows that the intensity of absorbance at 652 nm remained basically unchanged for five cycles indicating the DA-PPI nanozyme possess an eligible stability (Figure 3(d)).

**Figure 4:**
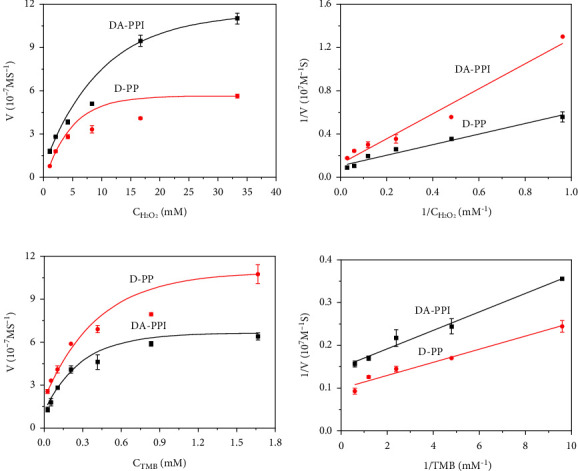
The enzyme kinetic analysis of D-PP nanozyme and DA-PPI nanozyme for H2O2 (a); the enzyme kinetic analysis of D-PP nanozyme and DA-PPI nanozyme for TMB (b); the double-reciprocal plot generated from Figure 4(a) (c); the double-reciprocal plot that generated from Figure 4(b) (d).

The catalytic process of the nanozyme fit for the Michaelis‒Menton kinetics [22]. The substrates include hydrogen peroxide and TMB. In order to measure the kinetic parameters of the DA-PPI nanozyme towards H_2_O_2_, the concentration of TMB is fixed first. The concentration of H_2_O_2_ is varied to detect and calculate the parameters of *K*_*m*_ and *V*_max_. The results are shown in Figure 4(a). As the concentration of H_2_O_2_ increases, the maximum reaction rate gradually approaches equilibrium. Combined with the double-reciprocal curve (Figure 4(b)), the relevant parameters of the catalytic efficiency of nanozymes for hydrogen peroxide are calculated and obtained. The results show that the affinity of DA-PPI nanozyme for hydrogen peroxide was better than that of D-PP nanozyme (4.51 mM vs. 9.28 mM, Table 1), but slightly less than that of graphene quantum dots (2.288 mM) and graphene oxide (2.301 mM) [23]. Similarly, the corresponding parameters of TMB were measured. The results are shown in Figures 4(c) and 4(d). The affinity of DA-PPI nanozyme for TMB was close to that of D-PP nanozyme (0.10 mM vs. 0.16 mM, Table 1). These results suggest that DA-PPI nanozyme exhibits a relative higher peroxidase-like activity than that of D-PP nanozyme.

### 3.3. The Intensity Assessment of Reactive Oxygen Species

**Figure 5:**
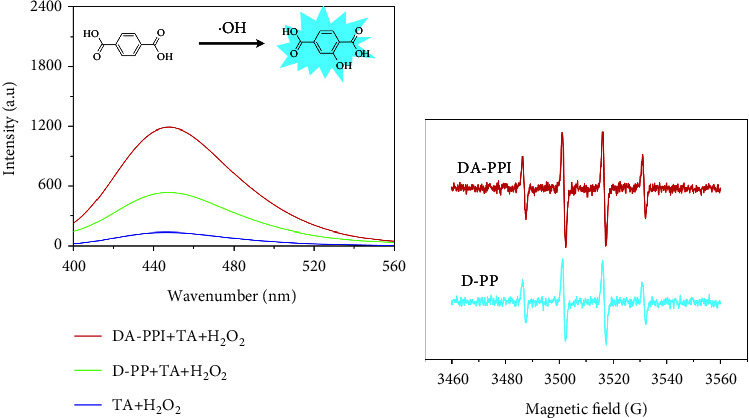
The fluorescence spectra of different samples (a). The ESR spectra of different samples with H_2_O_2_, DMPO and D-PP nanozyme and DA-PPI nanozyme (b).

In order to explain the high activity of DA-PPI nanozyme, terephthalic acid (TA) was employed to capture the hydroxyl radical in the system (photoluminescence (PL) method). The reaction product of TA and hydroxyl radical shows an obvious fluorescence emission at 450 nm [24]. The fluorescence intensity represents the amount of hydroxyl radicals in the system containing nanozyme and H_2_O_2_. In this part, 10 *μ*L of Pd-Pt-Ru solution was added to 2.5 mL of acetic acid buffer solution (HAC-NaAC pH = 4) containing 100 *μ*L of TA (0.05 mM) and 400 *μ*L H_2_O_2_ (62.50 mM). The fluorescence spectra of the samples were collected after 30 minutes of reaction by fluorescence spectroscopy (F-7100FL). The result is shown in Figure 5(a). It can be seen that the amount of hydroxyl radicals generated in the DA-PPI nanozyme group is significantly higher than that of the D-PP nanozyme group. Furthermore, the ESR was used to confirm the amount of hydroxyl radicals generated in the system of nanozyme and H_2_O_2_. The DMPO (5,5-dimethyl-1-pyrroline N-oxide) was employed to capture the hydroxyl radicals in the DA-PPI nanozyme and D-PP nanozyme group. The reaction product of DMPO and hydroxyl radical will produce a characteristic splitting peak of 1 : 2 : 2 : 1 [25]. The result is shown in Figure 5(b). The intensity of the splitting peak represents the amount of hydroxyl radicals in the system. It can be seen that the amount of hydroxyl radicals generated by the DA-PPI nanozyme group is significantly higher than that of the D-PP nanozyme group. The result is consistent with the PL methods. The high amount of hydroxyl radicals generated by the DA-PPI nanozyme group lead up to the high peroxidase-like activity.

**Figure 6:**
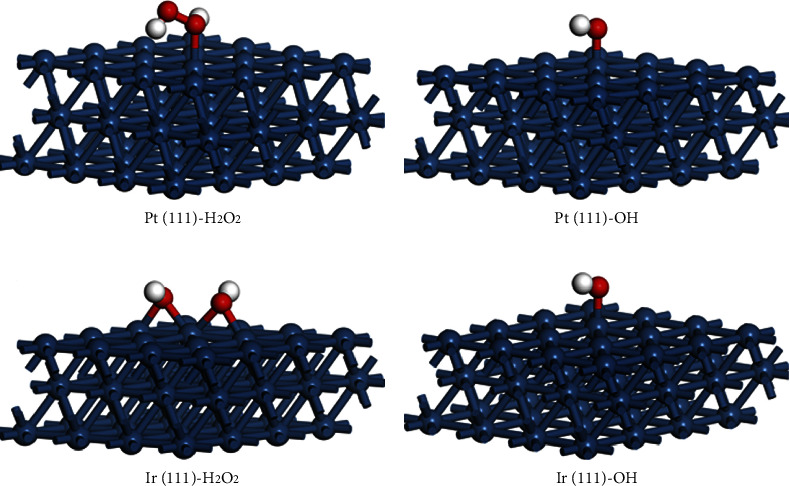
The calculation model of H_2_O_2_ on Pt (111) (a). The calculation model of OH on Pt (111) (b). The calculation model of H_2_O_2_ on Ir (111) (c). The calculation model of OH on Pt (111) (d).

The PL and ESR spectra present that DA-PPI nanozyme can produce a relative high amount hydroxyl radical with H_2_O_2_ participated. However, the reason is not clear. In this part, the density functional theory (DFT) was employed to analyze the adsorption energy of D-PP nanozyme and DA-PPI nanozyme to H_2_O_2_ and OH [26–28]. Figures 6(a) and 6(b) show the adsorption model of H_2_O_2_ and OH on Pt (111). The adsorption energy of H_2_O_2_ and OH on Pt (111) are −0.64 eV and −2.35 eV, respectively. Interestingly, the H_2_O_2_ decompose to OH on the optimized model of Ir (111) (Figure 6(c)). The adsorption energy of OH on Ir (111) is −2.69 eV (Figure 6(d)), which coincides to the optimal value of previous report [29]. The results indicate that the appropriate adsorption energy of OH on Ir (111) endows DA-PPI nanozyme a relative high peroxidase-like activity.

### 3.4. The Antimicrobial Activity of DA-PPI Nanozyme

**Figure 7:**
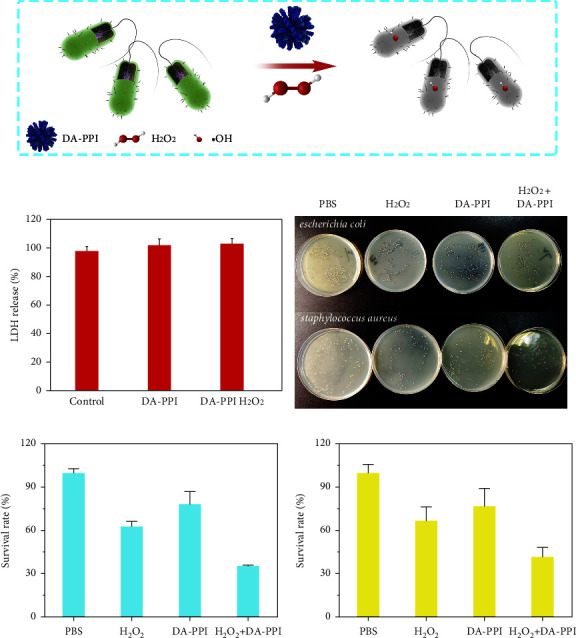
The possible antibacterial mechanism of DA-PPI nanozyme (a). LDH release after treatment 12 h in absence or presence of DA-PPI or DA-PPI with 0.5 mM H_2_O_2_ (b). Photographs of bacterial colonies of *E. coli* and *S*. *aureus* in different groups (c). The survival of *E. coli* in different groups (d). The survival of *S*. *aureus* in different groups (e).

It has been reported that some highly efficient nanozymes are capable of producing highly oxidative •OH by decomposing biosafety levels of H_2_O_2_ to defend bacterial infections without toxicity to healthy tissues. For example, the oxygenated-group-enriched carbon nanotubes and Au/g-C_3_N_4_ hybrid nanozymes exhibit excellent peroxidase activity to produce reactive oxygen species (ROS) and act as efficient antibacterial agents for actual wound disinfection with low concentration of H_2_O_2_ [30–32]. The cytotoxicity test was carried out in this part. The result shows that the DA-PPI nanozyme has minimal toxicity to human cells (Figure 7(b)). The antibacterial activities of DA-PPI nanozyme against *E. coli* and *S. aureus* were then evaluated by measuring the survival ratios in our study. As presented in Figures 7(c)–7(e), when the bacteria were incubated with DA-PPI nanozyme, the bacterial survival ratios of *E. coli* and *S. aureus* were 82.39% ± 13.00% and 77.03% ± 12.17%, respectively, which show little difference between the PBS and DA-PPI nanozyme groups, indicating that the antibacterial effect of DA-PPI nanozyme on bacteria was limited. Meanwhile, the survival ratios of *E. coli* and *S. aureus* in the DA-PPI nanozyme + H_2_O_2_ group were 35.5% ± 2.15% and 41.85% ± 6.49% and 61.81% ± 3.80% or 67.10% ± 9.19% in the H_2_O_2_ group, which means that the DA-PPI nanozyme can catalyze low concentration of H_2_O_2_ to produce high level of hydroxyl radicals, which can damage proteins, lipids, and DNA of microbial cells; then, the bacteria were killed and eliminated (Figure 7). Although H_2_O_2_ alone could kill the bacteria without DA-PPI nanozyme, the relatively higher concentration of H_2_O_2_ was required to effectively kill bacteria. Moreover, the high concentration of H_2_O_2_ will injure the normal tissues. Therefore, DA-PPI nanozyme could enhance the antibacterial activity of H_2_O_2_ due to its high peroxidase-like activity (Figure 7(a)).

## 4. Conclusion

**Scheme 1:**
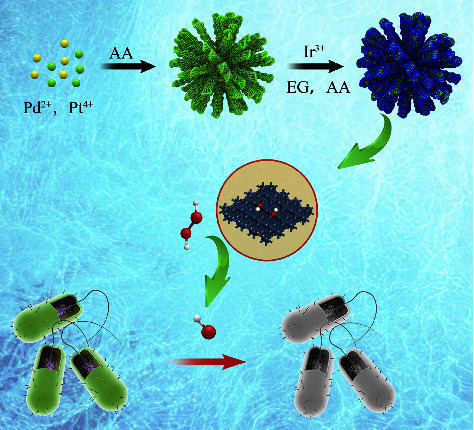
The possible antimicrobial mechanism of DA-PPI nanozyme.

In this work, Ir was employed to deposit on the surface of Pd-Pd nanodendritic to form DA-PPI nanozyme. The DA-PPI nanozyme was characterized by SEM, TEM, TEM-EDX, and XPS. The DA-PPI nanozyme possessed a higher peroxidase-like activity compared to D-PP nanozyme. The *K*_*m*_ of DA-PPI nanozyme to H_2_O_2_ is 2.06 times than that of D-PP nanozyme. According to the DFT calculation, the high peroxidase-like activity is attributed to the appropriate adsorption of Ir (111) to OH. Finally, the obtained DA-PPI nanozyme inhibits the growth of *E. coli* and *S. aureus* through peroxidase-like activity, which shows promise for overcoming the critical challenges in the treatment of bacterial infection (Scheme 1).

## Figures and Tables

**Table 1:** The enzyme kinetic parameters of D-PP nanozyme and DA-PPI nanozyme.

Catalyst	Substrate	*K*_*m*_ (mM)	*V*_max_ (M s^−1^ × 10^−7^)	Reference
DA-PPI nanozyme	H_2_O_2_	4.51	9.29	This work
	TMB	0.10	5.83	

D-PP nanozyme	H_2_O_2_	9.28	8.03	This work
	TMB	0.16	10.15	

HRP	H_2_O_2_	3.70	0.87	32
	TMB	0.43	1.00	

Fe_3_O_4_	H_2_O_2_	154	0.98	32
	TMB	0.098	0.34	

GQDs	H_2_O_2_	2.29	1.56	23
	TMB	—	—	

GO	H_2_O_2_	2.30	0.20	23
	TMB	—	—	

## Data Availability

The data used to support the findings of this study are included within the article and are available from the corresponding authors upon request.
